# A Comprehensive Look at the Development of Asthma in Children

**DOI:** 10.3390/children11050581

**Published:** 2024-05-11

**Authors:** Ileana Diana Diaconu, Veronica Gheorman, Gabriela Adriana Grigorie, Cristian Gheonea, Tiberiu-Stefanita Tenea-Cojan, Beatrice Mahler, Ion Alexandru Voropanov, Mihnea Cristian Firoiu, Andreea Silvia Pîrvu, Alexandru Bogdan Popescu, Renata Văruț

**Affiliations:** 1 Department of Pediatric Pneumology, University of Medicine and Pharmacy of Craiova, Petru Rareș 2 Str., 200349 Craiova, Romania; diana.diaconu@umfcv.ro; 2 Department of Medical Semiology, University of Medicine and Pharmacy of Craiova, Petru Rareș 2 Str., 200349 Craiova, Romania; 3 Department of Pneumology, University of Medicine and Pharmacy of Craiova, Petru Rareș 2 Str., 200349 Craiova, Romania; gabriela.grigorie@umfcv.ro; 4 Department of Pediatrics, University of Medicine and Pharmacy of Craiova, Petru Rareș 2 Str., 200349 Craiova, Romania; cristian.gheonea@umfcv.ro; 5 Department of Surgery, University of Medicine and Pharmacy of Craiova, CFR Hospital of Craiova, Stirbei-Voda Str., 200374 Craiova, Romania; tiberiu.tenea@umfcv.ro; 6 Department of Pneumology, Faculty of Medicine “Carol Davila”, “Marius Nasta” Institute of Pneumoftiziology, 050159 Bucharest, Romania; beatricemahler@umfcd.ro; 7 Department of Pediatric Pneumology, Carol Davila University of Medicine and Pharmacy, “Marius Nasta” Institute of Pneumoftiziology, 050159 Bucharest, Romania; ion-alexandru.voropanov@drd.umfcd.ro; 8 Department of Urology, Fundeni Clinical Institute, Carol Davila University of Medicine and Pharmacy, Sos. Fundeni nr. 258, 022328 Bucharest, Romania; dr.mihneafiroiu@gmail.com; 9 Department of Biochemistry, University of Medicine and Pharmacy of Craiova, 200349 Craiova, Romania; andreea.pirvu@umfcv.ro; 10 Radiology Department, Targoviste County Emergency Hospital, Tudor Vladimirescu 48 Str., 130083 Targoviste, Romania; dr.popescubogdan@gmail.com; 11 Department of Pharmacology, University of Medicine and Pharmacy, Petru Rareş Street 2-4, 200349 Craiova, Romania; renata.varut@umfcv.ro

**Keywords:** childhood asthma, genetic predisposition, environmental factors, early-life influences, prenatal care, preventive measures, respiratory health, allergen exposure, maternal smoking, immunomodulation

## Abstract

Asthma, a prevalent chronic respiratory condition affecting millions of children globally, presents a significant health challenge. This review critically examines the developmental pathways of asthma in children, focusing on genetic, environmental, and early-life determinants. Specifically, we explore the impact of prenatal and postnatal factors such as maternal smoking, nutrition, respiratory infections, and allergen exposure on asthma development. Our analysis highlights the intricate interplay of these influences and their contribution to childhood asthma. Moreover, we emphasize targeted strategies and interventions to mitigate its burden, including genetic counseling for at-risk families, environmental modifications to reduce triggers, and early-life immunomodulation. By delving into these preventive measures and interventions, our review aims to provide actionable insights for healthcare professionals in developing tailored strategies to address the complexities of childhood asthma. In summary, this article offers a detailed examination of asthma development in children, aiming to enhance understanding and inform efforts to reduce its burden through targeted interventions.

## 1. Introduction

In recent years, the prevalence of asthma in children has underscored its significance as a major public health challenge [1]. As a chronic noncommunicable disease, asthma not only affects the well-being of individual children but also imposes a substantial burden on society [2]. The repercussions span a spectrum from heightened mortality and morbidity to disruptions in education, manifesting as lost school days for children and productivity setbacks due to parental workdays lost [2,3,4]. The economic implications, coupled with the compromise in the quality of life for those affected, highlight the urgency of comprehensively understanding the factors that contribute to the development, severity, and long-term outcomes of asthma in the pediatric population.

Epidemiological studies have been instrumental in unraveling the complexities of asthma, tracking its prevalence across diverse populations and shedding light on environmental influences that shape its trajectory [5,6,7].

Recent epidemiological studies indicate that the incidence of childhood asthma has shown complex patterns over recent decades, with factors such as environmental changes and diagnostic shifts influencing these trends.

The incidence of childhood asthma has been linked to various environmental and genetic factors, suggesting an interaction between genes and environmental conditions such as exposure to pollutants, lifestyle changes and reduced microbial diversity. These factors are thought to have contributed to the increase in asthma cases seen from the late 20th century into the early 21st century [8].

Globally, the incidence of asthma tends to vary by age group and over time, with the highest incidence reported in very young children (0–4 years) and generally decreasing with age. However, the incidence has shown a slight increase over time in the 0–9-year-old age group. Interestingly, the most recent birth cohorts have exhibited higher asthma incidence rates, although mortality from asthma has generally decreased over the same periods, particularly in older age groups [9].

Another study highlights that asthma incidence in childhood has varied, with certain birth cohorts and geographical regions experiencing different rates of asthma incidence. Factors contributing to these variations include racial, sex, and socio-economic differences, as well as differing environmental exposures [10].

Overall, while the global burden of asthma in children shows some decrease in mortality due to better management and treatments, the incidence remains high, particularly in younger age groups, underscoring the need for ongoing monitoring and preventive strategies.

While early life factors, including genetic predisposition, have emerged as key players in asthma onset, the intricacies of its causation remain elusive. Recent strides in genetic research and advancements in epidemiological methodologies offer a glimpse into a more nuanced understanding of asthma, promising insights into its origins and potential avenues for intervention [11].

The multitude of factors at play in the development of asthma underscores the need for a comprehensive approach to understanding its intricacies in children [12]. This review aims to delve into the various aspects of asthma development in children, from early-life factors to environmental influences, in order to shed light on this pressing public health issue. By taking a comprehensive look at the development of asthma in children, we can work towards a better understanding of its origins and potential strategies for prevention and management.

In our review process, we initially conducted comprehensive searches using databases such as PubMed, Scopus, and Web of Science, utilizing relevant keywords such as “asthma development in children”, “pediatric asthma pathogenesis”, “asthma risk factors” and “preventive strategies”. We focused on articles published in peer-reviewed journals within the last two decades, considering seminal studies and landmark papers regardless of their publication date.

The retrieved articles were then meticulously screened based on title, abstract, and full-text review to ensure alignment with our review objectives. We prioritized articles that directly addressed pediatric asthma development, encompassing epidemiological trends, genetic susceptibility, environmental exposures, early-life factors, immune dysregulation, and preventive interventions. Articles were excluded if they did not meet our inclusion criteria or if they did not provide relevant insights into asthma development in pediatric populations.

The selection criteria for our narrative review include:

Relevance to Asthma Development: Prioritizing articles directly addressing the development of asthma in children, including epidemiological trends, genetic susceptibility, environmental exposures, early-life factors, immune dysregulation, and preventive interventions.

Quality and Credibility: Selecting articles from reputable peer-reviewed journals with rigorous editorial standards and high-quality research methodologies to ensure reliability and credibility.

Diversity of Perspectives: Including articles representing diverse perspectives and research methodologies, such as epidemiological studies, genetic analyses, environmental research, immunological investigations, and clinical trials, to provide a comprehensive overview of asthma development.

Recent and Relevant Literature: Prioritizing recent publications reflecting the current state of knowledge in pediatric asthma research, as well as seminal studies and landmark papers contributing significantly to our understanding of asthma development in children.

Consistency with Research Objectives: Evaluating each selected article based on its alignment with the objectives of our narrative review, which aim to synthesize existing evidence on asthma development and identify key factors influencing disease susceptibility and outcomes in pediatric populations.

## 2. Relevant Sections

### 2.1. The Role of Genetic Predisposition in Childhood Asthma Development

**Figure 1:**
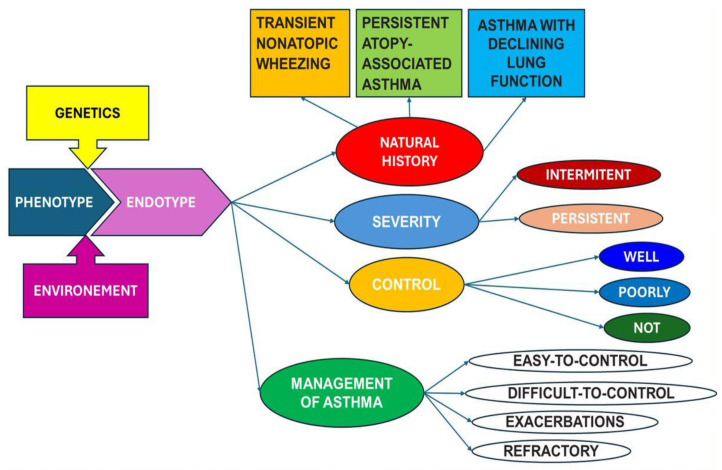
Evolutionary patterns of childhood asthma based on natural evolution and response to management from phenotype to endotype.

The role of genetic predisposition in the development of childhood asthma has been a focal point of extensive research, aiming to unravel the intricate interplay between genetic factors and environmental influences contributing to the onset of this prevalent respiratory condition (Figure 1). Asthma, characterized by chronic inflammation of the airways and heightened airway responsiveness, is recognized as a complex and multifactorial disease, where genetic predisposition plays a pivotal role, particularly during a child’s formative years [13].

Numerous studies have consistently demonstrated a substantial familial aggregation of asthma, indicating a hereditary component in its development [14,15,16,17]. Individuals with a family history of asthma are more likely to exhibit symptoms and receive a diagnosis, underscoring the presence of a genetic predisposition [14]. The heritability of asthma, estimated through twin and family studies, ranges widely from 35% to 95%, signifying the significant influence of genetic factors on susceptibility to the disease [18].

Researchers have made strides in identifying specific genes associated with asthma susceptibility [19,20,21]. These genes are integral to various biological processes, including immune regulation, airway smooth muscle function, and inflammatory responses. Notable examples include genes encoding cytokines, receptors, and proteins involved in the regulation of the immune system. Variations or mutations in these genes can contribute to an individual’s predisposition to asthma and influence the severity of the condition [21].

The role of genetic predisposition in childhood asthma development is a complex and multifaceted aspect of research, with numerous studies shedding light on the genetic factors contributing to disease susceptibility and progression. Wang and colleagues employed a Mendelian randomization approach to identify potential drug targets for the treatment of allergic diseases, utilizing genome-wide association study (GWAS) data from Ferreira et al. [22]. They further validated their findings using GWAS data from the FinnGen and UK Biobank cohorts, conducting sensitivity tests and protein–protein interaction analyses to elucidate the underlying mechanisms. Their study provides valuable insights into the genetic basis of allergic diseases and offers potential therapeutic avenues for intervention.

Similarly, Jakwerth et al. conducted a genome-wide transcriptome analysis in children, focusing on the impact of the 17q21 genotype (SNP rs72163891) on mucosal GSDMB expression—a gene associated with asthma/wheeze phenotype [23]. Their findings revealed that the 17q21 risk allele enhances mucosal GSDMB expression in a genotype- and phenotype-dependent manner, correlating with the activation of pro-inflammatory and immune response pathways. This study highlights the intricate interplay between genetic variations and immune dysregulation in childhood asthma pathogenesis.

Furthermore, Maison et al. conducted a multicenter study on a cohort of patients followed for 2 years, categorizing them into four phenotypes based on blood eosinophil counts and allergen-specific serum IgE antibodies [24]. Their findings suggest a direct onset of asthma, with the T2-high phenotype characterized by excessive production of specific IgE to allergens and increased production of IL-5—a key cytokine involved in eosinophilic inflammation. Importantly, this phenotype was observed across all ages, indicating early origins of T2-high asthma and emphasizing the importance of early intervention strategies in childhood asthma management.

Together, these studies underscore the significant role of genetic predisposition in childhood asthma development and provide valuable insights into the underlying mechanisms driving disease pathogenesis. Understanding the genetic determinants of asthma susceptibility and progression holds promise for the development of targeted therapeutic approaches aimed at mitigating disease burden and improving outcomes in pediatric populations.

The complexity of genetic contributions to asthma becomes apparent in the polygenic nature of the disease [25]. Unlike monogenic disorders caused by a single gene mutation, asthma results from the cumulative effects of multiple genetic variations. This polygenic inheritance pattern implies that there is no single “asthma gene” but rather a combination of genetic factors that collectively contribute to an individual’s susceptibility.

Furthermore, the role of epigenetics in asthma development has gained attention. Epigenetic modifications, such as DNA methylation and histone acetylation, can influence gene expression without altering the underlying DNA sequence [26]. These modifications are responsive to environmental exposures, suggesting a dynamic interplay between genetic predisposition and environmental factors in asthma pathogenesis.

**Figure 2:**
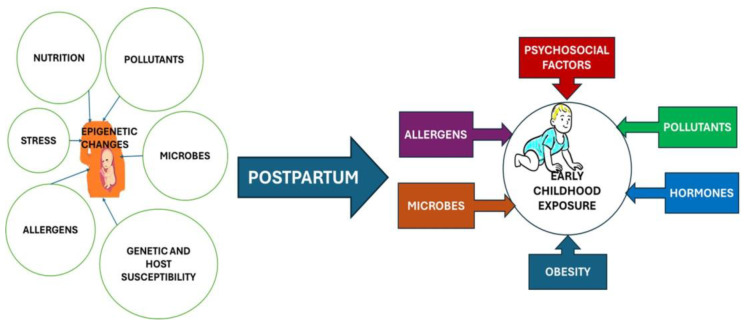
The nexus of genetic predisposition and environmental influences.

The interaction between genetic predisposition and environmental exposures is a critical aspect of childhood asthma development (Figure 2) [27]. Environmental factors, including exposure to allergens, air pollutants, respiratory infections, and tobacco smoke, can act as triggers for asthma in genetically susceptible individuals. For instance, a child with a genetic predisposition to asthma may be more susceptible to developing the condition when exposed to environmental allergens like dust mites or pet dander [28,29].

**Figure 3:**
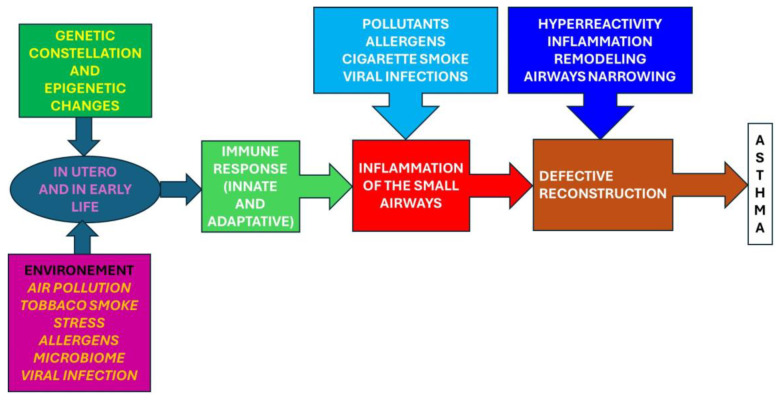
Exploring the genetic constellation: unraveling epigenetic changes.

Understanding the genetic basis of childhood asthma has practical implications for both prevention and treatment (Figure 3). Identifying individuals with a heightened genetic risk can enable targeted interventions, such as environmental modifications to reduce exposure to known triggers [30,31]. Additionally, advancements in pharmacogenomics may pave the way for personalized treatment approaches, tailoring asthma medications based on an individual’s genetic profile to optimize therapeutic outcomes.

Targeted Environmental Interventions:

1. Use of Allergen-Impermeable Bedding Covers:

Description: Covers that prevent allergens like dust mites from penetrating pillows and mattresses.

Effectiveness: Studies have shown that using allergen-impermeable bedding can significantly reduce dust mite exposure and is associated with improved respiratory symptoms in children with asthma who are allergic to dust mites [32].

2. Removing Carpets:

Description: Replacement of carpeted floors with hard surfaces to minimize the accumulation of pet dander, dust mites, and other allergens.

Effectiveness: Research indicates that removing carpets, especially in the bedroom and other areas where children spend a lot of time, can reduce allergen levels and asthma symptoms [33].

3. Maintaining Low Indoor Humidity:

Description: Keeping indoor humidity levels below 50% to inhibit the growth of dust mites and mold.

Effectiveness: Lower humidity levels have been associated with decreased dust mite populations and mold growth, potentially reducing asthma exacerbations in sensitized individuals [34].

4. Reducing Exposure to Pet Dander:

Description: Measures include keeping pets out of the bedroom, regular pet bathing, and using high-efficiency particulate air (HEPA) filters.

Effectiveness: These interventions have been found to reduce levels of pet allergens in the home and may help in controlling asthma symptoms in children allergic to pets [35].

5. Use of Air Purifiers:

Description: Utilizing HEPA filters in air purifiers to reduce airborne allergens.

Effectiveness: Air purifiers can lower the concentration of airborne allergens such as pollen, pet dander, and dust mite allergens, contributing to better asthma control [36].

While these interventions can effectively reduce allergen exposure and alleviate symptoms in individuals with diagnosed asthma, further research is needed to determine their role in preventing asthma development in genetically predisposed individuals. It is essential to recognize the distinction between managing symptoms in allergic asthmatics and preventing the onset of asthma in at-risk populations.

In conclusion, the role of genetic predisposition in childhood asthma development is a multifaceted and dynamic aspect of this complex respiratory condition. While genetics significantly contribute to susceptibility, the interaction with environmental factors adds layers of complexity to the disease’s etiology.

### 2.2. Childhood Asthma: Insights from Gut–Skin–Lung Axis and Atopic March

The gut–skin–lung axis suggests a connection between the microbial environments of the gut, skin, and respiratory tract. This axis highlights how disturbances in one area can affect the others:

Gut Microbiome: The gut microbiome plays a crucial role in immune system development. Early alterations in gut bacteria, potentially due to factors like antibiotic use or diet, can predispose children to inflammatory conditions like asthma. The gut microbiome affects systemic immune responses, which can influence respiratory health.

Skin Barrier: Skin acts as a barrier and an immune organ. Disruptions in the skin barrier, such as those seen in eczema, can lead to increased exposure to allergens. These allergens can sensitize the immune system, leading to responses in other areas, including the lungs.

Lung Exposure: The respiratory tract is directly exposed to the external environment, making it a critical site for initiating allergic responses. Inhalation of allergens can trigger immune responses that may be exacerbated by existing sensitizations from gut- and skin-related disturbances.

Atopic March

The concept of the atopic march describes the typical progression of allergic diseases, where early-life eczema may lead to food allergies, followed by asthma and allergic rhinitis:

Early Onset: It often starts with eczema (atopic dermatitis) in infancy.

Progression: Children with eczema are at higher risk of developing food allergies, asthma, and allergic rhinitis later in childhood.

Immunological Links: The progression is thought to be due to the development of a skewed immune response towards an allergic type (Th2 responses), which is influenced by genetic and environmental factors, including those affecting the gut–skin–lung axis.

Understanding the interactions within the gut–skin–lung axis and the mechanisms of the atopic march can help in developing preventive strategies and treatments for childhood asthma and other allergic diseases. This might include interventions aimed at maintaining a healthy gut microbiome through diet or probiotics, strategies to strengthen the skin barrier, and approaches to reduce early allergen exposure in susceptible children [37,38,39].

### 2.3. The Impact of Environmental Factors, Such as Pollution and Allergens, on Asthma Onset in Children/on Asthma Development

The impact of environmental factors on asthma onset in children is multifaceted, encompassing various elements that interact in complex ways [40,41,42].

Outdoor air pollutants, such as particulate matter (PM), nitrogen dioxide (NO_2_), sulfur dioxide (SO_2_), and ozone (O_3_), have been implicated in the development and exacerbation of asthma symptoms in susceptible individuals. Among these pollutants, particulate matter is of particular concern due to its diverse sources and variable particle sizes, each posing unique risks to respiratory health.

Particulate matter, or PM, refers to a mixture of solid particles and liquid droplets suspended in the air, categorized based on their size as PM10 (particles with a diameter of 10 μm or less) and PM2.5 (particles with a diameter of 2.5 μm or less). These particles can originate from various sources, including vehicle emissions, industrial processes, construction activities, and natural sources like wildfires and dust storms [43].

PM10 particles, although larger in size, can still penetrate deep into the respiratory tract and reach the lungs, where they can trigger inflammation and exacerbate asthma symptoms. PM2.5 particles, on the other hand, are even smaller and can penetrate farther into the lungs, reaching the alveoli and potentially entering the bloodstream. Their smaller size allows them to evade the body’s natural defense mechanisms, leading to more severe respiratory and cardiovascular effects.

Exposure to PM, particularly PM2.5, has been associated with an increased risk of asthma development and exacerbations in children. Prolonged exposure to elevated levels of PM2.5 has been linked to reduced lung-function growth, increased airway inflammation, and higher rates of asthma-related hospitalizations and emergency department visits [44].

Moreover, the composition of particulate matter can vary depending on its source, with certain components, such as black carbon, heavy metals, and organic compounds, posing additional health risks. For example, black carbon, a component of diesel exhaust, has been shown to induce oxidative stress and inflammation in the airways, exacerbating asthma symptoms in children.

The impact of outdoor air pollutants, including particulate matter, on asthma onset and exacerbations in children is significant. Understanding the different particle sizes and their sources allows for targeted interventions to mitigate exposure and reduce the burden of asthma on respiratory health. Public health efforts aimed at reducing emissions and improving air-quality standards are crucial for protecting children from the adverse effects of outdoor air pollution on asthma and overall respiratory health.

### 2.4. Investigating Early-Life Influencess, Including Prenatal and Postanatal Factors, in the Development of Asthma in Children

Asthma development in children is influenced by a myriad of factors, with both prenatal and postnatal exposures playing crucial roles in shaping respiratory health. Understanding these early-life influences is essential for unraveling the complexities of asthma etiology and implementing effective preventive strategies.

#### 2.4.1. Prenatal Factors

Maternal Smoking:

Maternal smoking during pregnancy is a well-established prenatal risk factor for childhood asthma. Exposure to tobacco smoke in utero can lead to impaired lung development, altered immune responses, and increased susceptibility to respiratory infections, all of which contribute to the development of asthma in childhood [45]. Additionally, maternal smoking may induce epigenetic changes in the developing fetus, further increasing the risk of asthma later in life [46].

Maternal Diet:

Emerging evidence suggests that maternal diet during pregnancy may influence asthma development in offspring. A diet rich in fruits, vegetables, and omega-3 fatty acids has been associated with a lower risk of childhood asthma, possibly due to its anti-inflammatory properties and beneficial effects on immune regulation [47]. Conversely, a diet high in processed foods, sugary beverages, and saturated fats may increase the risk of asthma in children, potentially through mechanisms involving oxidative stress and inflammation [48].

Maternal Stress:

Prenatal maternal stress has been identified as a potential risk factor for childhood asthma. Maternal stress during pregnancy can lead to dysregulation of the maternal–fetal hypothalamic–pituitary–adrenal (HPA) axis, resulting in altered immune responses and increased susceptibility to allergic diseases in offspring [49]. Animal studies have shown that prenatal stress can program the fetal immune system towards a pro-inflammatory phenotype, predisposing offspring to asthma-like symptoms [50].

Maternal Allergies and Asthma:

Maternal history of allergies and asthma is a significant predictor of childhood asthma. Genetic predisposition, combined with shared environmental exposures, contributes to an increased risk of asthma in offspring born to mothers with allergic diseases [51]. Maternal atopy can influence fetal immune development and alter the intrauterine environment, increasing the likelihood of allergic sensitization and asthma development in children [52].

#### 2.4.2. Postnatal Factors

Early-Life Respiratory Infections:

Respiratory infections during infancy, particularly those caused by respiratory syncytial virus (RSV) and rhinovirus, have been implicated in the development of childhood asthma. Severe viral respiratory infections in early life can trigger airway inflammation, disrupt lung development, and promote aberrant immune responses, setting the stage for asthma development in susceptible individuals [53]. Longitudinal studies have shown that children with a history of severe respiratory infections in infancy are at increased risk of developing asthma later in childhood [54].

Exposure to Allergens:

Early-life exposure to allergens, such as house dust mites, pet dander, and mold, plays a crucial role in asthma development. Sensitization to allergens during infancy can lead to the development of allergic asthma, characterized by airway inflammation and hyperresponsiveness [55]. Reducing allergen exposure in the home environment, through measures such as allergen-proof bedding, air purifiers, and pet avoidance, has been shown to reduce asthma symptoms and medication use in children with allergic asthma [55].

House dust mites, in particular, are pivotal contributors to allergic sensitization in childhood [56]. Exposure to house dust mites, which are commonly found in bedding, upholstery, and carpets, can lead to allergic reactions in susceptible individuals, including wheezing, coughing, and shortness of breath. Sensitization to house dust mites during infancy or early childhood increases the risk of developing allergic asthma later in life. Therefore, efforts to reduce exposure to house dust mites through environmental control measures such as using allergen-proof bedding, vacuuming regularly, and maintaining low humidity levels in the home are essential for preventing the development and exacerbation of asthma in children.

Breastfeeding:

Breastfeeding has long been recognized for its protective effects on respiratory health in infancy. Breast milk contains immunomodulatory factors such as immunoglobulins, cytokines, and growth factors that support the development of the infant’s immune system and provide protection against respiratory infections [57].

Early Antibiotic Use:

Early and frequent antibiotic use in infancy has been associated with an increased risk of childhood asthma. Antibiotics can alter the composition of the gut microbiome, disrupt immune development, and promote dysregulated inflammatory responses, all of which may contribute to asthma susceptibility [58]. Longitudinal cohort studies have shown that children exposed to antibiotics in early life are at higher risk of developing asthma later in childhood [59].

Impact of Diet:

Dietary factors have been increasingly recognized for their potential role in asthma development and exacerbations in children. High-fat diets, particularly those rich in saturated fats, have been associated with increased airway inflammation and asthma risk in children [60]. Conversely, diets rich in polyunsaturated fatty acids (PUFAs), such as omega-3 and omega-6 fatty acids, have been linked to reduced airway inflammation and improved lung function in children with asthma [61]. Additionally, vitamin D deficiency has been implicated in asthma pathogenesis, with supplementation showing potential benefits in reducing asthma exacerbations and improving asthma control in children [62]. The impact of diet on asthma risk is complex and multifactorial, involving interactions between dietary components, immune responses and genetic predisposition.

Obesity:

Obesity has emerged as a significant postnatal factor associated with childhood asthma development. Obese children are at increased risk of developing asthma, possibly due to systemic inflammation, altered adipokine levels, and mechanical effects of excess adipose tissue on lung function [63]. Longitudinal studies have shown a positive association between obesity in childhood and the incidence of asthma, highlighting the importance of addressing obesity as a modifiable risk factor for asthma prevention [64]. Moreover, recent evidence indicates that obesity may induce a type 2-like proinflammatory respiratory pattern in non-asthmatic adolescents, further emphasizing the intricate relationship between obesity and airway inflammation [65].

Early-life influences, including prenatal and postnatal factors, play a critical role in shaping the risk of childhood asthma. Recognizing the complex interplay between genetic predisposition, environmental exposures, and early-life events is essential for developing targeted preventive strategies aimed at reducing the burden of childhood asthma. It is important to note that, while interventions may effectively reduce symptoms in allergic asthmatics, true prevention of the disease requires addressing underlying risk factors before asthma develops. Therefore, our focus is on developing strategies that not only manage symptoms but also aim to prevent the onset of asthma in susceptible individuals. One such strategy with significant potential is allergen-specific immunotherapy (AIT). AIT aims to modulate the immune system’s response to allergens, addressing the root cause of allergic diseases like asthma.

Potential of Allergen-Specific Immunotherapy:

Allergen-specific immunotherapy (AIT) has emerged as a promising strategy for preventing the progression of allergic diseases, including asthma, in children. AIT involves the administration of gradually increasing doses of allergen extracts to induce immune tolerance and reduce allergic responses [66]. Several clinical trials and observational studies have demonstrated the efficacy of AIT in reducing asthma symptoms, medication use, and asthma exacerbations in children with allergic asthma [67]. By targeting the underlying allergic sensitization, AIT has the potential to modify the natural course of asthma and prevent its progression to a more severe phenotype [66,67].

Building upon the discussion of early-life influences and the potential of allergen-specific immunotherapy (AIT) in preventing childhood asthma, recent research findings shed light on the therapeutic implications of targeting specific immune pathways. A study by Musiol et al. delves into the role of TGF-β in allergic airway inflammation (AAI) and its modulation through AIT [68]. This study underscores the significance of TGF-β in driving effector T cell responses while also influencing the differentiation of regulatory T cells (Tregs). By elucidating the intricate mechanisms underlying AAI and the therapeutic potential of TGF-β modulation, this research further supports the rationale behind incorporating AIT into preventive strategies for childhood asthma. Recognizing the pivotal role of immune dysregulation in asthma pathogenesis, interventions targeting immune pathways such as TGF-β hold promise in mitigating disease progression and reducing the burden of asthma in susceptible individuals.

In addition to allergen-specific immunotherapy (AIT), it is essential to consider the potential of antibody-based therapy concepts in addressing pediatric asthma development. These therapies offer a targeted approach by focusing on specific antibodies associated with allergic reactions or inflammatory pathways involved in asthma pathogenesis [69]. By precisely targeting key molecules such as cytokines or immunoglobulins, antibody-based therapies aim to modulate immune responses and reduce airway inflammation, thus mitigating asthma symptoms and potentially preventing disease progression in children. Emphasizing the importance of exploring and developing such innovative therapeutic approaches could lead to more effective management strategies and improved outcomes for pediatric asthma patients.

### 2.5. The Potential for Preventive Measures and Interventions Based on a Comprehensive Understanding of Asthma Development in Children

A comprehensive understanding of the multifactorial nature of asthma development in children opens avenues for targeted preventive measures and interventions. By addressing genetic predisposition, environmental exposures and early-life influences, healthcare professionals and caregivers can collaboratively work towards minimizing the burden of childhood asthma.

#### 2.5.1. Genetic Counseling and Early Identification

Identification of High-Risk Individuals: Genetic screening and identification of children with a familial predisposition to asthma can facilitate early interventions. High-risk individuals can be closely monitored and preventive measures can be implemented from an early age.

The goal of genetic counseling in the context of asthma is to provide individuals and families with information about their genetic predisposition to the condition and to help them understand the implications of this risk. Genetic counseling aims to empower individuals to make informed decisions about their healthcare, including preventive measures to reduce the risk or severity of asthma symptoms.

Preventive measures that could be implemented for high-risk individuals identified through genetic screening include:

Environmental Modification: Genetic counseling may involve educating families about environmental factors that can trigger asthma symptoms, such as exposure to allergens (e.g., dust mites, pet dander, pollen), air pollution, tobacco smoke, and respiratory infections [70]. Implementing measures to minimize exposure to these triggers, such as using air purifiers, maintaining a clean indoor environment, and avoiding smoking, can help reduce the risk of asthma exacerbations.

Lifestyle Modifications: Encouraging healthy lifestyle habits such as regular exercise, balanced nutrition, and maintaining a healthy weight can contribute to overall respiratory health and potentially reduce the risk of asthma development or severity [71]. Genetic counseling can provide guidance on lifestyle modifications tailored to the individual’s genetic risk profile and family history.

Allergen Avoidance: For individuals with known allergen sensitivities identified through genetic screening or clinical history, allergen avoidance strategies may be recommended. This could include measures such as using allergen-proof bedding covers, minimizing exposure to pets, and avoiding outdoor activities during high-pollen seasons.

Medication Management: Genetic counseling may involve discussions about the appropriate use of asthma medications, including controller medications to prevent asthma symptoms and rescue medications for symptom relief. High-risk individuals may benefit from early initiation of controller medications under the guidance of healthcare providers to maintain optimal asthma control and reduce the risk of exacerbations.

Immunomodulators: In some cases, early intervention with immunomodulatory treatments like immunotherapy might be considered for high-risk individuals. Immunotherapy has shown effectiveness in altering the progression of allergic diseases, including asthma, particularly when initiated at a young age [72]. However, it is important to note that the decision to initiate immunotherapy should be based on clinical criteria and guidelines rather than genetic background alone. Healthcare providers should assess each patient’s clinical history, symptoms, and allergen sensitivities before recommending immunotherapy, in accordance with established guidelines and best practices in allergy and asthma management.

Vaccinations: Ensuring vaccination against respiratory infections such as influenza and pneumonia, which can exacerbate asthma or trigger its onset, can help prevent infections that are particularly severe in children at high risk for asthma [73].

Regular Monitoring and Follow-up: Genetic counseling provides an opportunity for individuals and families to understand the importance of regular monitoring for asthma symptoms and respiratory function. High-risk individuals may be encouraged to undergo periodic lung-function tests such as spirometry and to maintain regular follow-up appointments with healthcare providers for ongoing assessment and management.

Overall, the goal of genetic counseling in the context of asthma is to empower individuals and families to take proactive steps to mitigate the risk of asthma development or exacerbations.

Implementing these preventive measures in children identified as high risk through genetic screening can help manage or potentially reduce the impact of asthma. These strategies focus on modifying environmental, dietary, and immune factors that contribute to the disease’s development and severity [74].

#### 2.5.2. Prenatal Care and Education

Maternal Smoking Cessation Programs: Implementing targeted programs to assist pregnant women in quitting smoking can significantly reduce the risk of asthma in their children. Education on the detrimental effects of prenatal smoking and resources for smoking cessation can be integrated into routine prenatal care [75].

Nutritional Guidance: Offering nutritional guidance to pregnant women can promote a diet rich in fruits, vegetables, and omega-3 fatty acids, potentially mitigating the risk of childhood asthma. Educating expectant mothers on the impact of maternal diet on fetal development is crucial [76].

#### 2.5.3. Environmental Modifications

Indoor Air-Quality Improvement: Implementing measures to improve indoor air quality, such as reducing exposure to tobacco smoke, using air purifiers, and minimizing allergen sources, can have a significant impact on asthma prevention. Public health campaigns can raise awareness about the importance of a clean indoor environment [77].

Reduction in Outdoor Air Pollution: Implementing policies to reduce outdoor air pollution, especially in urban areas, can contribute to lowering the overall asthma risk in children. This may involve stricter regulations on industrial emissions, promotion of public transportation, and green urban planning [42,78].

#### 2.5.4. Early-Life Immunomodulation

Promotion of Breastfeeding: Encouraging and supporting breastfeeding, considering its potential immunomodulatory effects, can be part of preventive strategies. Public health initiatives can focus on educating mothers about the benefits of breastfeeding and addressing barriers to breastfeeding.

Probiotic and Microbiome Interventions: Research exploring the role of the gut microbiome in asthma development suggests that interventions such as probiotic supplementation might influence immune system maturation [79].

Recent studies continue to explore the effects of probiotic supplementation on asthma prevention in infants but have not demonstrated a significant protective benefit. A systematic review published in Pediatrics explored the impact of early probiotic supplementation (specifically Lactobacillus rhamnosus GG) during the first 6 months of life in high-risk infants but found no significant reduction in the cumulative incidence of asthma [80]. Similarly, another review focused on probiotic supplementation for eczema and asthma prevention, and it also failed to show a protective effect against asthma [81].

Furthermore, a systematic review examined multiple randomized controlled trials concerning probiotics’ effects on asthma and found that while probiotics did not prevent asthma, they might improve asthma control and pulmonary function in infants who already have asthma. However, the studies reviewed were mixed, and the benefits were not universally observed, indicating a need for more focused research to confirm these potential effects [82].

These findings collectively suggest that while probiotics may offer some benefits in managing existing asthma symptoms, their use as a preventive measure against the development of asthma in infants does not currently have robust support in the scientific literature. Further research is needed to explore potential benefits in asthma control and to identify if specific probiotic strains might be more effective.

#### 2.5.5. Vitamin Supplementation and Asthma Development in Children

Emerging evidence has indicated potential associations between certain vitamins and asthma risk, although the exact nature of these relationships remains under investigation.

Vitamin D has garnered attention for its potential impact on asthma development in children. While some studies suggest that low levels of vitamin D may increase the risk of asthma, the evidence is not entirely conclusive. Further investigation is needed to elucidate the precise role of vitamin D in asthma pathogenesis. Additionally, the effectiveness of vitamin D supplementation during pregnancy or early childhood in reducing asthma risk is still being explored [83,84].

Similarly, vitamin E, known for its antioxidant properties, has been investigated for its potential role in asthma prevention. Some studies suggest that higher dietary intake of vitamin E may be associated with a lower asthma risk in children, though definitive conclusions are lacking [85,86].

Vitamin C, another antioxidant, has also been studied for its potential impact on asthma risk. Research suggests that higher dietary intake of vitamin C may be linked to a reduced risk of asthma in children, although further studies are needed to confirm these findings [87,88].

The use of multivitamin supplements, which contain a combination of vitamins and minerals, is common. However, evidence regarding their effect on asthma risk is limited and inconsistent. While some studies suggest a potential protective effect of multivitamin supplementation against asthma development in children, additional research is necessary to establish clear conclusions [89,90].

In summary, while there is growing interest in the potential role of vitamins in asthma prevention, the current evidence is varied and inconclusive. Further research is warranted to better understand the impact of vitamins on asthma risk and to inform preventive strategies effectively.

#### 2.5.6. Educational Programs for Caregivers

Caregiver Education on Early Symptoms: Providing caregivers with information on recognizing early symptoms of asthma can lead to prompt medical intervention. Educational programs can empower parents and caregivers to monitor respiratory health and seek medical attention when necessary.

Allergen Avoidance Strategies: Educating caregivers on allergen avoidance strategies, such as proper cleaning practices and minimizing exposure to common allergens, can be instrumental in preventing allergic sensitization and subsequent asthma development.

#### 2.5.7. Access to Healthcare

Early Diagnosis and Management: Ensuring access to healthcare for all children regardless of socio-economic status is essential for early diagnosis and effective management of asthma. Timely intervention can prevent exacerbations and long-term complications.

Asthma Action Plans: Developing and implementing asthma action plans for children diagnosed with asthma equips caregivers and school personnel with clear guidelines for managing asthma symptoms, thereby reducing the impact of the condition on the child’s daily life.

In conclusion, a holistic approach to asthma prevention in children involves a combination of genetic awareness, prenatal care, environmental modifications, early-life immunomodulation, caregiver education, and improved access to healthcare. By implementing these preventive measures and interventions, the goal is not only to reduce the prevalence of childhood asthma but also to enhance the overall respiratory health and quality of life for children at risk.

## 3. Discussion

1. The Gut Microbiota and Asthma Risk

The gut microbiota plays a crucial role in immune system development and regulation, particularly during early life [91]. Disturbances in the establishment of a diverse and balanced gut microbiota, such as antibiotic use, cesarean section delivery, and formula feeding, have been associated with an increased risk of asthma in children. Conversely, exposure to beneficial microbes such as certain strains of *Bifidobacteria* and *Lactobacilli* through factors like breastfeeding and early-life environmental exposures may confer protection against asthma development.

2. Immune Dysregulation in Asthma

Asthma is characterized by aberrant immune responses, including Th2-mediated inflammation and airway hyperreactivity. Regulatory T cells (Tregs) play a crucial role in maintaining immune tolerance and suppressing excessive inflammation. Dysregulation of Treg function has been implicated in asthma pathogenesis, with evidence suggesting that alterations in gut microbiota composition may influence Treg development and function.

3. Microbiota-Immune Interactions in Asthma

The gut microbiota interacts with the host immune system through various mechanisms, influencing immune cell maturation, cytokine production, and inflammatory responses. Disruptions in this delicate balance can lead to immune dysregulation and contribute to the development or exacerbation of asthma. For example, alterations in gut microbiota composition may influence the production of short-chain fatty acids (SCFAs), which have immunomodulatory properties and can impact airway inflammation.

4. Epithelial Mucous Membrane Dysfunction and Asthma

The epithelial mucous membrane lining the respiratory tract serves as a physical barrier against environmental insults and pathogens. Dysfunction of the epithelial barrier, characterized by increased permeability and impaired mucociliary clearance, has been observed in individuals with asthma. Disruptions in epithelial barrier integrity may allow for the translocation of allergens, toxins, and microbial components into the underlying tissue, triggering immune responses and airway inflammation [92].

5. Therapeutic Strategies Targeting the Gut Microbiota in Asthma

Understanding the link between gut microbiota dysbiosis, epithelial mucous membrane dysfunction, immune dysregulation, and asthma has prompted interest in therapeutic interventions aimed at modulating the gut microbiota to prevent or manage asthma symptoms.

The gut microbiota, epithelial mucous membrane integrity, and immune system play integral roles in the pathogenesis of childhood asthma. Disruptions in gut microbiota composition, epithelial barrier function, and immune regulation during critical periods of immune system development may contribute to asthma susceptibility and severity. Strategies aimed at restoring gut microbiota balance, preserving epithelial barrier integrity, and modulating immune responses hold potential as preventive and therapeutic approaches for childhood asthma.

The Global Initiative for Asthma, Global Strategy for Asthma Management and Prevention, 2022, implement the idea that the onset and development of asthma result from the interaction between individual genetic predisposition and environmental factors [93]. This interaction occurring during intrauterine life as well as in the early days of extrauterine life represents an important link that we currently do not have clear and efficient means to nullify.

Many studies targeting the explanation of asthma onset and progression in children have focused on demonstrating the involvement of nutritional factors, allergic terrain, polluted climate, infectious agents, medication administration, and psychosocial context [93].

Although there are studies regarding the relationship between maternal diet during pregnancy and the onset of asthma in children, these have not been convincing enough. Thus, currently, changing the diet during pregnancy is not recommended for preventing asthma in children. Similarly, studies attempting to correlate maternal obesity or weight gain with the onset of asthma in children have not been convincing enough to recommend weight loss programs during pregnancy [93]. Breastfeeding as well as delayed introduction of solid foods have not proven to have notable effects in preventing asthma onset in children.

Supplementation of the diet with vitamin D, fish oil, long-chain polyunsaturated fatty acids, and probiotic administration has been studied in clinical trials, systematic reviews, and meta-analyses, but the results are controversial and do not provide clarification regarding their role and effectiveness in preventing childhood asthma onset.

The comprehensive understanding of immune, inflammatory, and genetic factors in asthma pathophysiology holds paramount clinical relevance, profoundly impacting various facets of clinical practice. This knowledge not only advances our comprehension of the disease’s mechanisms but also shapes the approach to diagnosis, treatment, and overall patient care.

### 3.1. Enhancing Asthma Diagnosis through Biomarker Integration

Clinicians can incorporate biomarkers associated with these factors to enhance the accuracy and specificity of asthma diagnosis, aiding in distinguishing asthma from other respiratory conditions with overlapping symptoms and ensuring timely and appropriate interventions. While fractional exhaled nitric oxide (FeNO) is incorporated into asthma diagnostic criteria and is a well-established biomarker for eosinophilic inflammation, the clinical application of genetic testing for asthma is still evolving. Biomarkers such as FeNO and eosinophil counts can indicate airway inflammation, which is a hallmark of asthma. Integrating these biomarkers into diagnostic protocols can help clinicians differentiate asthma from other respiratory conditions with similar symptoms, leading to more precise and timely interventions.

### 3.2. Optimizing Treatment Approaches

The identification of immune dysregulation, chronic inflammation, and genetic factors underscores the importance of tailored treatment approaches for asthma. While targeted therapies hold promise for addressing specific aspects of asthma pathophysiology, it is essential to acknowledge the role of environmental factors in asthma management. Therefore, alongside personalized medicine considerations, clinicians should emphasize environmental modifications as integral components of asthma treatment strategies.

### 3.3. Stratified Medicine

Stratifying patients based on their immune, inflammatory, and genetic profiles enables the development of more precise and effective treatment regimens. Subgroups with distinct pathophysiological features can be identified, guiding clinicians in selecting therapies that align with the underlying mechanisms driving their asthma. This approach contributes to improved treatment outcomes and a more patient-centered care model.

### 3.4. Predictive Medicine

A deeper understanding of genetic factors opens avenues for predictive medicine in asthma. Genetic markers can serve as indicators of disease susceptibility and progression [94]. Early identification of at-risk individuals allows for proactive interventions, such as targeted environmental modifications or preventive pharmacotherapy, ultimately reducing the likelihood of asthma development or exacerbations.

Targeted environmental modifications refer to specific changes or interventions aimed at modifying the environmental factors known to trigger or exacerbate asthma symptoms in individuals with a genetic predisposition to the condition. These modifications are tailored to the individual’s genetic risk profile and may include measures such as:

Allergen Reduction: dentifying and minimizing exposure to allergens that are known to trigger asthma symptoms, such as dust mites, pet dander, pollen, mold, and cockroach allergens. This may involve using allergen-proof bedding covers and air purifiers, vacuuming frequently, and keeping indoor humidity levels low to prevent mold growth.

Air-Quality Improvement: Measures can be implemented to improve indoor air quality, such as reducing exposure to environmental pollutants (e.g., cigarette smoke, volatile organic compounds) and ensuring proper ventilation in indoor spaces. This could involve avoiding smoking indoors, using air filters or purifiers, and reducing the use of chemical cleaners and fragrances.

Avoidance of Respiratory Irritants: Individuals can be educated about avoiding exposure to respiratory irritants, such as strong odors, air pollutants, and cold air, which can trigger asthma symptoms or exacerbations. This may include recommendations to minimize exposure to outdoor air pollution, irritant chemicals, and respiratory infections.

Healthy Lifestyle Promotion: Individuals should be encouraged to adopt a healthy lifestyle, including regular exercise, balanced nutrition, adequate hydration, and stress management. These lifestyle modifications can support overall respiratory health and potentially reduce the risk of asthma development or exacerbations.

Preventive pharmacotherapy involves the proactive use of medications to reduce the frequency and severity of asthma exacerbations in individuals already diagnosed with the condition, particularly those at risk due to genetic factors. These medications, including long-term controller medications such as inhaled corticosteroids (ICS), leukotriene modifiers, and long-acting beta-agonists (LABAs), aim to minimize airway inflammation and hyperresponsiveness, thereby controlling asthma symptoms and preventing exacerbations when taken regularly as prescribed.

Biological agents, also known as biologicals, target specific inflammatory pathways and are used as a therapeutic strategy. While they do not prevent the initial development of asthma, they play a crucial role in managing the disease and reducing exacerbation risk in individuals with severe asthma or specific immune phenotypes. Similarly, allergen immunotherapy is used proactively in individuals with allergic asthma to desensitize the immune system and reduce allergic reactions over time, ultimately decreasing asthma symptoms and medication requirements.

### 3.5. Enhancing Patient Care

Knowledge of immune, inflammatory, and genetic factors enhances overall patient care by fostering a holistic and individualized approach. Clinicians can engage in informed discussions with patients about the nature of their asthma, potential triggers, and personalized management strategies. This not only improves patient education but also promotes active patient participation in their care, leading to better adherence to treatment plans.

### 3.6. Guiding Long-Term Management

The integration of immune, inflammatory, and genetic information guides long-term management strategies. Clinicians can anticipate potential challenges, such as exacerbations related to specific triggers or a predisposition to persistent inflammation. This foresight allows for proactive adjustments in treatment plans and frequent monitoring, contributing to better disease control and improved quality of life for patients.

The comprehensive understanding of immune, inflammatory, and genetic factors in asthma pathophysiology transcends theoretical knowledge, profoundly influencing clinical practice. From refined diagnostics to personalized treatment strategies and enhanced patient care, this knowledge empowers clinicians to navigate the complexities of asthma with precision and efficacy, ultimately improving outcomes for individuals living with this chronic respiratory condition.

## 4. Conclusions

In conclusion, the pathophysiology of asthma in children is a nuanced interplay of immune dysregulation, inflammation, and genetic factors. The manifestation of asthma often entails a chronic inflammatory response and heightened airway sensitivity, culminating in recurrent wheezing and breathlessness that significantly impact a child’s quality of life. Recognizing the intricate mechanisms underlying these processes is paramount for devising effective treatment and management strategies.

Environmental factors emerge as crucial triggers in this context, with allergens and pollutants playing pivotal roles in exacerbating asthma symptoms. Understanding and mitigating these triggers become instrumental for healthcare professionals and parents alike, fostering an environment conducive to the respiratory well-being of children with asthma.

While a definitive cure remains elusive, a spectrum of preventive measures and treatment options empowers the management of asthma in children. Strategies encompass steering clear of known triggers, leveraging medications for symptom control and prevention, and embracing lifestyle modifications like regular physical activity and a nutritious diet. Importantly, the key lies in early detection and intervention, paving the way for improved outcomes by facilitating enhanced symptom control and optimizing respiratory function.

In navigating the complexities of childhood asthma, a holistic approach that integrates medical intervention, environmental awareness and proactive lifestyle choices stands as the cornerstone. By advancing our understanding of asthma’s underlying mechanisms and fostering a collaborative effort between healthcare providers and caregivers, we can collectively strive towards ensuring a healthier, more comfortable life for children grappling with asthma.

### Future Directions

First, future research should focus on identifying the genetic and environmental factors that contribute to the development of asthma in children. By understanding the underlying causes of asthma, we can develop targeted prevention strategies and personalized treatment approaches. Genetic studies can help identify susceptibility genes, while environmental research can help identify triggers and risk factors.

Second, more emphasis should be placed on early detection and diagnosis of asthma in children. The earlier asthma is diagnosed, the sooner appropriate interventions and management plans can be implemented to control symptoms and prevent exacerbations. New diagnostic tools and screening methods should be developed to improve early detection and accuracy of asthma diagnosis in children.

Third, there should be a greater focus on understanding the impact of asthma on children’s quality of life and long-term outcomes. Research should investigate the psychological, social, and emotional effects of living with asthma, as well as the potential long-term complications and comorbidities associated with the condition. This knowledge can help guide the development of comprehensive care plans that address the holistic needs of children with asthma.

Fourth, a future direction in the study of childhood asthma involves exploring the impact of socioeconomic disparities on asthma prevalence and outcomes. Children from low-income families and disadvantaged communities are disproportionately affected by asthma, and it is crucial to understand the underlying social and environmental determinants that contribute to these disparities. By addressing social determinants of health and implementing policies to reduce environmental exposures in these communities, we can work towards reducing the burden of childhood asthma in vulnerable populations.

Fifth, future research should explore novel treatment options and interventions for managing asthma in children. This includes investigating new medications, delivery devices, and therapeutic approaches that are safe, effective, and well tolerated in pediatric populations. Additionally, research should assess the benefits of non-pharmacological interventions such as lifestyle modifications, environmental control measures, and alternative therapies.

Finally, efforts should be made to implement comprehensive asthma management programs in various healthcare settings, including primary care, schools, and community-based clinics. These programs should emphasize patient education, asthma action plans, and self-management strategies to empower children and their families to better control their asthma. Additionally, integrating multidisciplinary care teams and leveraging technology for remote monitoring and telemedicine can improve access to specialized asthma care for children in underserved areas.

In conclusion, a comprehensive look at the development of asthma in children requires a multi-faceted approach that addresses genetic, environmental, diagnostic, quality-of-life, treatment, and healthcare delivery aspects. By pursuing these future directions, we can hope to improve the prevention, diagnosis, management, and outcomes of asthma in children, ultimately enhancing their overall health and well-being and reducing the burden of this respiratory disease.

## Data Availability

The data presented in this study are available in article.
